# Tandem mass tag-based serum proteomic profiling revealed diabetic foot ulcer pathogenesis and potential therapeutic targets

**DOI:** 10.1080/21655979.2022.2027173

**Published:** 2022-01-22

**Authors:** Xiao-Ting Yu, Feng Wang, Jia-Tong Ding, Bo Cai, Juan-Juan Xing, Guang-Hua Guo, Fei Guo

**Affiliations:** a Burns Institute, the First Affiliated Hospital of Nanchang University, NanChang, JiangXi, China; b Ningbo Institute for Medicine & Biomedical Engineering Combined Innovation, Ningbo Medical Centre Lihuili Hospital, Ningbo University, Ningbo, Zhejiang, China

**Keywords:** Diabetic foot ulcer, serum, proteomics, TMT

## Abstract

Diabetic foot ulcer (DFU), one of the most serious complications of diabetes mellitus, is associated with a high amputation rate and decreased life quality. The impact of blood serum proteins on the occurrence and development of DFU has attracted a lot of interest. In this study, we aimed to define and compare the serum proteome of patients with DFU and healthy control (HC) to provide new insights into DFU pathogenesis. DFU patients and age- and sex-matched HCs were enrolled in this study (n = 54). We screened alterations in blood serum proteins from DFU patients and HC using a tandem mass tag (TMT) method based on liquid chromatography-mass spectrometry (LC-MS/MS) quantitative proteomics, and the differentially expressed proteins (DEPs) were further validated by parallel reaction monitoring (PRM) and enzyme-linked immunosorbent assay (ELISA). A total of 173 DEPs (100 up-regulated and 73 down-regulated) were identified between the DFU and HC groups (*P* < 0.05). Proteomic and bioinformatics analyses indicated that the proteins in the DFU group were mainly related to extracellular matrix (ECM)-receptor interaction and complement and coagulation cascades. The up-regulated DEPs were further verified by PRM and ELISA. LRG1, CD5L, CRP, IGHA1, and LBP were proved upregulated in DFU and these proteins are mainly related to immune response and complement activation. Our findings help to provide a more comprehensive understanding of the pathogenesis of DFU and new insight into potential therapeutic targets.

## Introduction

Diabetes mellitus is an increasing public health issue that around half a billion people worldwide live with the disease [1]. Given the high rate of microvascular and macrovascular complications in patients with diabetes over their lifetime [2], diabetes mellitus has a significant economic impact on patients, families, and health systems. Diabetes mellitus leads to complications such as foot ulcers, coronary heart disease, stroke, chronic kidney disease, and neuropathy [3,4]. Among these complications, diabetic foot ulcer (DFU) has been described as the costliest diabetes-specific complications due to prolonged and recurrent hospitalizations, infections, and gangrene [2,5]. The lifetime incidence of DFU in diabetic patients is estimated at 19% – 34% [6]. According to a report from the International Diabetes Federation, 9.1 to 26.1 million people will develop DFU annually [6]. Moreover, approximately 20% of DFU with moderate or severe infections resulted in various levels of amputation [7]. Early detection and treatment help to decrease the chances of amputation [8].

The pathophysiological mechanisms underlying the development of DFU have been proposed [9,10]. Uncontrolled blood glucose levels are mostly recognized as the factor initiating the pathological processes in foot soft tissue abnormalities [11]. High blood glucose concentrations lead to the formation of advanced glycation end-products, and the increased levels of these end-products inhibit wound healing [12]. Moreover, a previous study showed that exogenous C-reactive protein (CRP) and serum amyloid A contributed to increasing inflammation and inducing the formation of vascular networks [13]. Circulating levels of lipoprotein-associated phospholipase A2 and interleukin-18 are associated with an increased Wagner grade [14]. Taken together, the role of serum proteins in the pathogenesis of DFU is being increasingly recognized. However, to our knowledge, the global analysis of serum proteins in patients with DFU has never been performed.

Proteomic analysis enables the identification of molecular mechanisms responsible for the development of a specific disease in a high-throughput, accurate, sensitive, and repeatable manner [15]. The tandem mass tag (TMT)-liquid chromatography-tandem mass spectrometric (LC-MS/MS) method has been well-developed to identify and quantify proteins [16]. In this study, we aimed to profile and quantify differentially expressed serum proteins (DEPs) between DFU patients and healthy controls (HC) using TMT technology and provide new insight for understanding the fundamental pathological processes underlying diabetic ulcers using bioinformatics approaches. We hope that our research will be informative for future studies into the occurrence, development, prevention, and therapeutic targets of DFU.

## Material and methods

### Recruitment of participants

The study protocol was approved by the Ethics Committee of the First Affiliated Hospital of Nanchang University (GF20180305). All participants provided signed informed consent. The participants were recruited from the First Affiliated Hospital of Nanchang University in Jiangxi, China, between August 2020 and September 2020. The exclusion criteria included neurodegenerative diseases, cardiovascular diseases, chronic viral infection, pressure ulcers, venous ulcers, arterial insufficiency ulcers, and other serious diseases. All subjects were nonsmokers and not alcohol-addicted. DFU was diagnosed according to the guidelines of the World Health Organization [17]. The inclusion/exclusion criteria are listed in Table 1. HC serum samples were collected from healthy individuals in the Department of Health Examination. Peripheral blood samples were drawn 12 h post-fasting. To eliminate inter-individual differences, equal amount of three serum samples from either DFU or HC group were randomly pooled into a tube as one sample [18].

**Table 1.** Inclusion/exclusion criteria

**Inclusion Criteria**
1. Patients aged≥18 years and <80 years
2. Primary diagnosis of DFU (base on the criteria of the WHO)
3. Written informed consent
**Exclusion criteria**
1. Neurodegenerative diseases (Parkinson’s disease, Alzheimer’s disease)
2. Cardiovascular disease (cardiac insufficiency, uncontrolled hypertension)
3. Chronic viral infection (viral hepatitis infection, HIV infection)
4. Pressure ulcers, venous ulcers and arterial insufficiency ulcers
5. Other serious diseases (malignancy, immunodeficiency)
6. Pregnant or lactating females
7. Unfavorable lifestyle habits (smoking, heavy alcohol consumption)

DFU: diabetic foot ulcer; WHO: World Health Organization.

### Protein extraction and digestion

The Agilent Multiple Affinity Removal Columns (4.6 × 100 mm) equipped in the HPLC systems (Agilent, USA) were used to deplete high-abundance proteins according to the manufacturer’s instructions [19,20]. The samples were prepared in sodium dithionite buffer (4% sodium dodecyl sulfate, 100 mM Tris-HCl, and 1 mM dithiothreitol, pH7.6). After the protein concentration was determined using the bicinchoninic acid (BCA) protein assay kit (Bio-Rad, USA), a filter-aided sample preparation procedure was applied to digest the above-qualified serum protein samples [16].

### TMT labeling

According to the protocol of the TMT labeling kit (Thermo Scientific, USA), the peptides were reconstituted in 0.1% (v/v) formic acid and every 100 μg of peptide mixture of each pooled sample was reconstituted and labeled with the TMT-labeling reagents [21].

### Mass spectrometry data analysis

The separated peptides were analyzed using LC-MS/MS on a Q Exactive mass spectrometer (Thermo Scientific). Mascot 2.2 and Proteome Discoverer 1.4 software was used for protein identification and quantification of the separated peptides. The average expression rates were defined as the fold-change (FC) compared to the average of the biological triplicates in the HC group. Proteins with a *P*-value < 0.05 and an FC >1.2 (up-regulated) or < 0.83 (down-regulated) were considered to be differentially expressed [22].

### Functional analysis of DEPs

Gene Ontology (GO) and Kyoto Encyclopedia of Genes and Genomes (KEGG) enrichment analyses were performed to investigate the biological functions of the DEPs. Using Blast2Go (https://www.blast2go.com/) software [23], the GO functions of the DEPs were evaluated for three GO categories: biological processes (BP), molecular function (MF), and cellular components (CC). Enriched signal and metabolism pathways were mapped using the online KEGG database with KEGG Automatic Annotation Server software. A two-tailed Fisher’s exact test was applied to analyze GO function and KEGG pathway enrichment of the DEPs, with a *P*-value of < 0.05 considered significant. The subcellular structure prediction software CELLO was used to predict subcellular protein localization.

### Gene set enrichment analysis

Gene set enrichment analysis (GSEA) was performed by uploaded the identified protein list of DFU and HC onto the online tool of OmicStudio (https://www.omicstudio.cn/tool). Associated biological functions of identified proteins with statistical significance were then obtained by the GSEA algorithms [24]. The gene sets of the BP were obtained from the Molecular Signature Database (MSigDB C5 databases, version 7). The criterion for statistical significance was set at a *P*-value of < 0.05 [25].

### Protein-protein interaction network construction

The protein-protein interaction (PPI) network was constructed using the string database (http://string-db.org/) and an interaction score of ≥1 was set to denote a significant interactive relationship. Then, we reconstructed and analyzed the PPI networks using the Cytoscape software (version 3.2.1) [26].

### PRM and ELISA

To verify the reliability of TMT results, the levels of selected proteins were further quantified by PRM analysis [27]. Skyline3.5.0 software was used to analyze the original PRM files and quantify the target proteins and peptides [22]. The leucine-rich repeat family of proteins (LRG1), CD5 molecule-like (CD5L), immunoglobulin heavy constant alpha 1 (IGHA1), lipopolysaccharide-binding protein (LBP), and C-reactive protein (CRP) ELISA kits were from Cusabio, China. ELISA was performed according to the instructions.

## Statistical analysis

Statistical analyses were performed using Graphprism 8.0. The data are expressed as the mean ± SEM. The independent sample t-test was used to compare the averages of the two groups, and a two-tailed Fisher’s test was applied to analyze GO and pathways. A *P*-value of < 0.05 was considered statistically significant.

## Results

### Baseline characteristics of the participants

The average age of the DFU group was 58.96 ± 2.57 years and 15 female and 12 male subjects were included. The average age of the HC was 61.87 ± 2.53 years and 15 female and 12 male subjects were included. There were no differences in age or gender (all *P* > 0.05). Full details are provided in Table 2.

**Table 2.** Characteristics of the participants

Characteristics	DFU (n = 27)	HC (n = 27)
Age (years)	58.96 ± 2.57	61.87 ± 2.53
Sex (F/M)	15/12	15/12
GLU (mmol/L)	9.72 ± 1.64*	5.12 ± 0.17
ALT (U/L)	13.64 ± 2.20	13.32 ± 1.69
AST (U/L)	19.68 ± 2.93	21.62 ± 1.58
TBIL (μmol/L)	7.21 ± 1.14	9.79 ± 1.59
CREA (μmol/L)	72.57 ± 8.23	68.64 ± 3.67
WBC (×10^9/L)	7.54 ± 0.92*	5.17 ± 0.36

GLU: glutamic acid; ALT: alanine aminotransferase; AST: aspartate aminotransferase; TBIL: total bilirubin; CREA: creatinine; WBC: white blood cells (Data were expressed as mean ± SEM, *P < 0.05 vs. HC).

### TMT-based proteomics identified 173 DEPs

**Figure 1.**
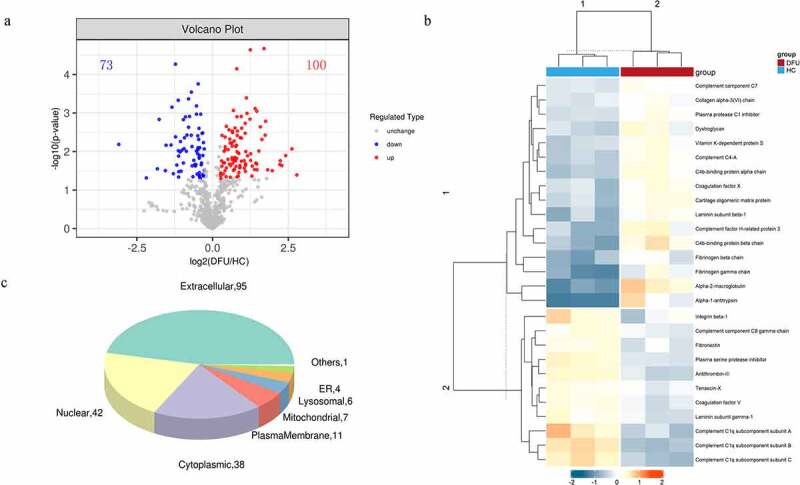
The DEPs between DFU patients and HC. (a) Volcano plot depicts the DEPs between DFU patients and HC. The X-axis represents log2 (DFU/HC), and the Y-axis represents the P-value of -log10. (b) DEPs involved in ECM-receptor interaction and the complement and coagulation cascades (rows) in DFU patients (red bars) and HC (blue bars). (c) Subcellular localization analysis of all DEPs was performed using the subcellular structure prediction software CELLO. The red and blue dots represent up-and down-regulated proteins respectively. DFU, diabetic foot ulcer; HC, healthy control.

We used TMT-based proteomics to identify the DEPs in the serum of DFU patients and HC to explore the potential mechanisms involving DFU pathogenesis. A total of 4065 peptides were identified in serum in this study (Supplementary File 1). And 647 non-redundant proteins were identified and quantified by Mascot 2.2 and Proteome Discoverer 1.4 software (Supplementary File 2). Only proteins and peptides shared between the DFU and HC samples were compared. One hundred and seventy-three DEPs were identified, including 73 down-regulated (FC < 0.83) and 100 up-regulated (FC > 1.2) with a *P*-value of <0.05 (Supplementary File 3). The identified DEGs were visualized by a volcano plot (Figure 1(a)).

### Protein cluster analysis indicates that the expression patterns of DFU is distinctive

We utilized a hierarchical clustering algorithm and generated a heatmap to visualize the dataset to figure out the related biological functions or biological process of the identified DEPs (Figure 1(b)). The expression patterns were very similar within each group. However, the patterns of DFU and HC are clearly different and can be separated effectively with the identified DEPs. The results indicated that the DEPs are mainly related to extracellular matrix (ECM)- receptor interaction and the complement and coagulation cascades.

### Bioinformatics analysis indicates the DEPs are mainly involved in ECM-receptor interaction, and the complement and coagulation cascades

**Figure 2.**
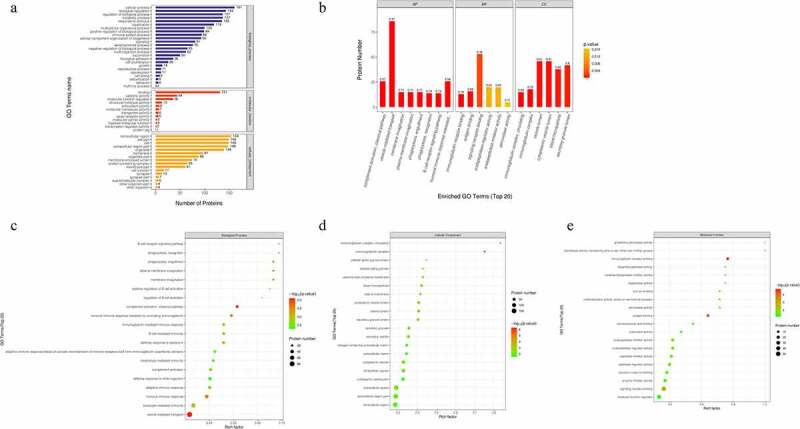
Gene Ontology (GO) term enrichment analysis of the DEPs. (a) The number of DEPs. The Y-axis represents the GO term name. (b) Enriched GO functional classifications. The Y-axis represents the number of DEPs under each functional classification. The color of the bar indicates the *P*-value. As the color changes from orange to red, the statistical significance changes from low to high. The label above the bar shows the enrichment factor (rich factor ≤ 1). (c-e) Enrichment factors. The Y-axis represents the number of DEPs in each functional category. The color of the bar indicates the *P*-value. As the color changes from green to red, the statistical significance changes from low to high.

To reveal the biological functions of the DEPs, subcellular localization and GO enrichment were performed. The subcellular localization analysis showed that the DEPs were widely distributed in the extracellular area (95 proteins), nuclear area (42 proteins), plasma membrane (11 proteins), mitochondria (7 proteins), lysosomes (6 proteins), endoplasmic reticulum (4 proteins), and others (1 protein) (Figure 1(c)). In GO functional analysis, these DEPs were found to participate in multiple foundational biological processes and enable various molecular functions (Figure 2(a)). The top 20 generally changed GO terms were compared (Figure 2(b)). The main BPs of the enriched DEPs were complement activation (classical pathway), vesicle-mediated transport, plasma membrane invagination, phagocytosis, and engulfment recognition (Figure 2(c)). The top CCs included both circulating and non-circulating immunoglobulin complexes (Figure 2(d)). In MF analysis, the DEPs were primarily enriched in immunoglobulin receptor binding, antigen binding, and signaling receptor binding (Figure 2(e)).

**Figure 3.**
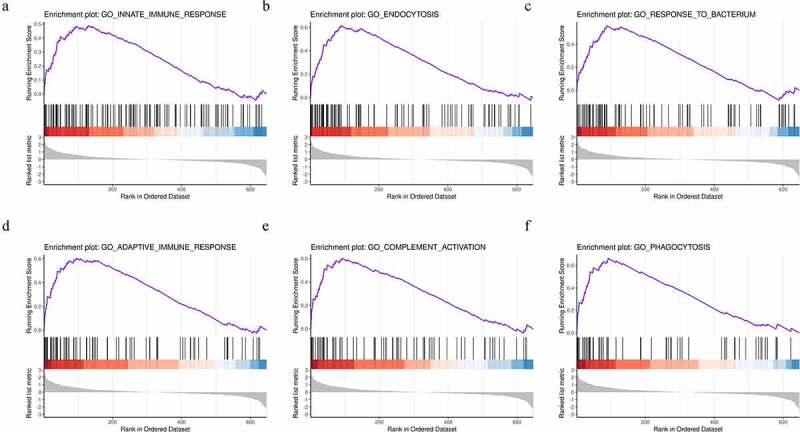
Identification of the top six enriched biological processes in DFU patients by gene set enrichment analysis (GSEA) analysis. (a) Innate immune response. (b) Endocytosis. (c) Response to bacterium. (d) Adaptive immune response. (e) Complement activation. (f) Phagocytosis.

To further explore the functional differences in protein expression between the DFU and HC groups, we performed GSEA analysis based on the BPs. The results showed that the DFU group was enriched in proteins regulating the innate immune response, endocytosis, the response to bacterium, the adaptive immune response, complement activation, and phagocytosis (Figure 3(a-f)). These enriched gene sets were closely correlated to the immune response and complement activation.

**Figure 4.**
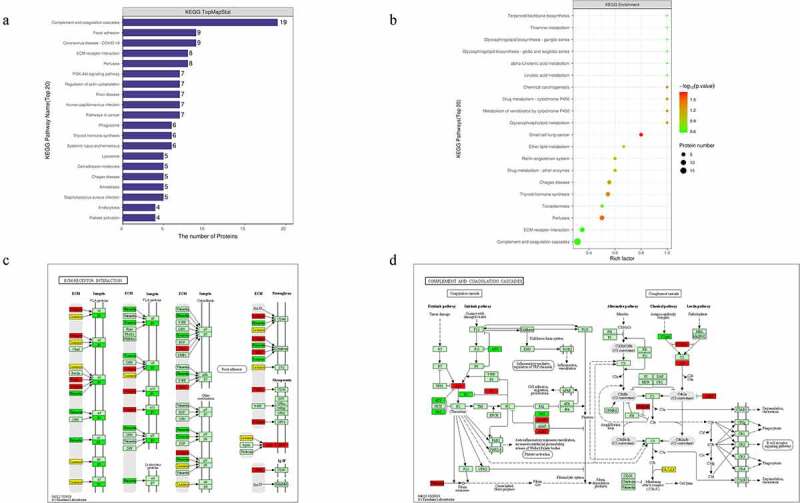
Signaling pathway enrichment analysis of the DEPs. (a) The number of DEPs. The Y-axis represents the KEGG pathway name. (b) Enrichment factors. The Y-axis represents the number of DEPs in each functional category. The color of the bar indicates the *P*-value. As the color changes from green to red, the statistical significance changes from low to high. (c-d) ECM-receptor interaction and complement and coagulation cascades were obtained from global proteome data by KEGG pathway analysis. Red and green graphics represent the up-regulated and down-regulated proteins, respectively, and the yellow graphics represent both up-and down-regulated DEPs.

Furthermore, KEGG enrichment analysis for DEPs suggested that the DEPs were mainly involved in extracellular matrix (ECM)-receptor interaction, and complement activation and coagulation cascades (Figure 4(a-b)). Among the ECM-related proteins, collagen VI alpha 3 (COL6A3), laminin subunit beta-1 (LAMB1), cartilage oligomeric matrix protein (COMP), and dystroglycan 1 (DAG1) were found to be commonly activated when we examined changes in DFU serum (Figure 4(c)). The complement and coagulation cascades-related proteins alpha-2-macroglobulin homolog (A2M), C4, C4b-binding protein (C4BP), and vitamin K-dependent protein S (PROS1) were also increased in DFU patient serum (Figure 4(d)).

### The PPI Network analysis results support the involvement of DEPs in ECM-receptor interaction and the complement and coagulation cascades

**Figure 5.**
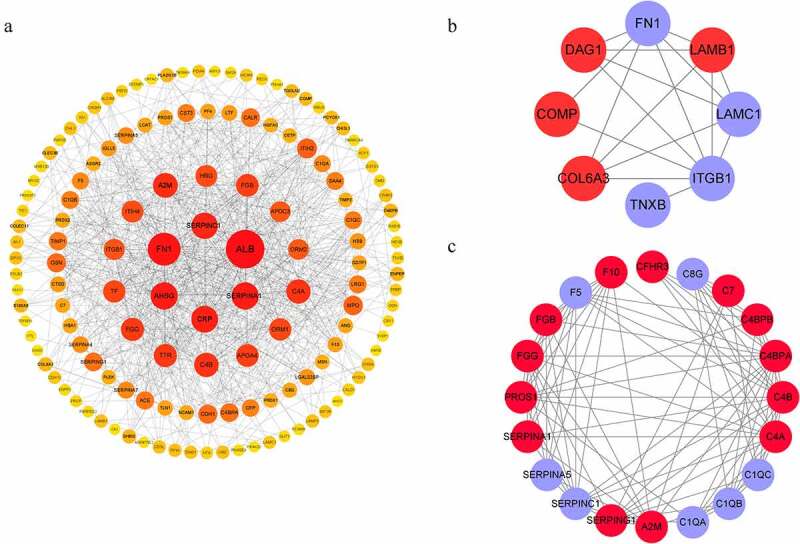
The interacted network of proteins analyzed by STRING. (a) All detected DEPs. (b) DEPs involved in ECM-receptor interaction. (c) DEPs involved in the complement and coagulation cascades. Red and blue graphics represent up-regulated and down-regulated proteins, respectively.

Based on GO and KEGG prediction data, we analyzed the protein interaction network of the DEPs (Figure 5(a)). PPI analysis showed that a considerable number of these proteins were associated with ECM-receptor interaction and the complement and coagulation cascades, which was consistent with the KEGG functional enrichment findings (Figure 5(b-c)).

### PRM and ELISA assays validate the increase of LRG1, CD5L, IGHA1, LBP, and CRP in DFU serum

**Figure 6.**
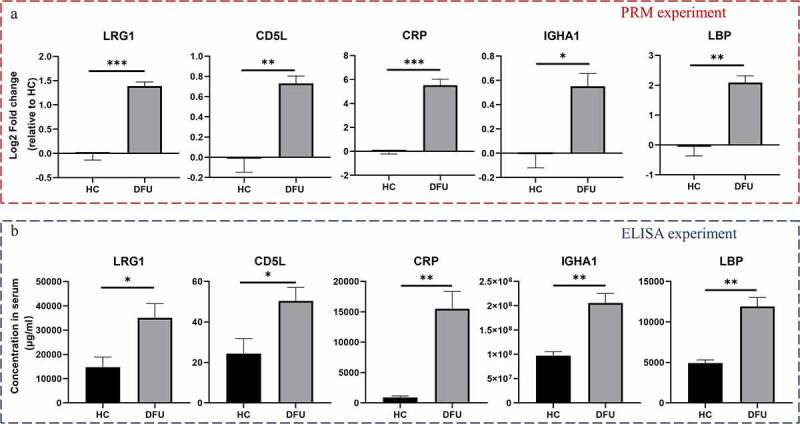
Validation of the DEPs. The levels of LRG1, CD5L, CRP, IGHA1, and LBP in DFU patient and HC serum were detected by PRM (a) and ELISA (b). Data are shown as the mean ± SEM, n = 3. *P < 0.05, ** *P* < 0.01, *** *P* < 0.001 vs. HC.

To validate the reliability of TMT-labeling of proteomic data, PRM analysis and ELISA were conducted. Based on the quantitative results from proteomics and bioinformatics analyses, we narrowed the number of proteins of interest to 14 selected proteins. Supplementary File 4 shows the Skyline analysis results of the target peptides. Among these validated proteins, LRG1, CD5L, IGHA1, LBP, and CRP were increased in DFU patient serum (*P* < 0.05) (Figure 6(a), Supplementary File 5). These results were further confirmed by ELISA (Figure 6(b)).

## Discussion

Our study strengthens the argument that serum components activate the complement and coagulation cascades via ECM-receptor interaction after tissue injury in diabetes. The DE molecules identified in DFU patient serum activate complex inflammatory networks and will serve as biomarkers of tissue injury or therapeutic targets.

The pathway analyses in this study showed that the DEPs in DFU patient serum were closely related to ECM-receptor interaction. ECM components and their fragments such as glycosaminoglycans and hyaluronan often act as damage-associated molecular patterns (DAMPs) during inflammatory processes, and interactions among these components and their cellular receptors are necessary for inflammatory processes in injury to the skin [28]. Subsequently, DAMPs or PAMPs detected by the soluble macromolecules via inflammatory fluid-phase pathways, rapidly activate the protein cascades containing the complete, and coagulation cascades [29,30], which are the main defense systems that mediate inflammation and thrombosis.

### ECM-activation in DFU

ECM components provide cells with physical support for adhesion and regulate proliferation, differentiation, and the fate of the cells [31]. The aberrant turnover of ECM components is often detected under inflammatory conditions [32]. The ECM-receptor interaction pathway and focal adhesion pathway were identified here. The interactions directly or indirectly lead to the control of adhesion, migration, differentiation, proliferation, and apoptosis [33]. LBP was validated as highly expressed in DFU patient serum, and this acute-phase protein initiates immune responses by binding to bacterial lipopolysaccharide (LPS) [34]. LBP is involved in the breakdown of the ECM by evoking the expression of matrix metalloproteinase. Another up-regulated protein was COMP. COMP plays a key role in bone and cartilage biology and processes including inflammation and angiogenesis [35]. Denton and colleagues reported that exogenous COMP may act as a paracrine factor to activate ERK1/2 signaling via CD36 to enhance adipogenesis [36]. CD36 and other cell-surface-associated components mediate specific interactions between cells and the ECM. CD5L secreted by macrophages was reported to circulate in the blood and interact with CD36. CD5L is correlated with the modulation of leukocyte migration [37] and macrophage recruitment [38]. Here, CD5L was validated as a serum protein significantly increased in DFU patient samples. Increased blood concentrations of CD5L have been observed in patients with a systemic autoimmune syndrome, and high serum levels of CD5L in patients were potentially related to disease activity [39].

### Activation of the complement and coagulation cascades found in DFU

The release of endogenous DAMPs, including mitochondrial or ECM peptides, nuclear, and cytosolic proteins from mechanically damaged or necrotic cells into the extracellular environment activate innate immunity. Here, we identified seven mitochondrial proteins, 42 nuclear proteins, and the above-mentioned ECM-related proteins in DFU patient serum, which can serve as or interact with DAMPs. The enriched proteins identified in the current study were implicated in neutrophil, monocyte, and B cell activity, highlighting a putative role for innate and humoral immunity in the pathogenesis of DFU.

Activation of the innate immune system, including complement activation and the recruitment and activation of neutrophils, is a key character of the early phase of tissue injury [40,41]. The complement system functions in the recognition and elimination of invading pathogens, and the removal of self-derived danger [42–44]. We identified the DEPs as A2M, C4A, C4B, C4BP, C7, and fibrinogen gamma chain (FGG), which were related to the complement system. Complement can be activated in DFU patients with these DEPs, leading to a series of proteolytic events, ultimately resulting in opsonization and lysis of the pathogens [45]. The main BPs of the enriched DEPs we identified were the classical pathway of complement activation, vesicle-mediated transport, plasma membrane invagination, phagocytosis, and engulfment recognition.

The recognition of DAMPs or PAMPs from microbial pathogens induces an inflammatory response. Higher circulating levels of LBP in the DFU samples were identified and quantified in this study. LBP is an acute-phase protein that binds to bacterial LPS to initiate immune responses [34]. Our findings support the role of another acute-phase protein, CRP, which activates the complement classical pathway, likely in response to PAMPs or DAMPs [46,47] during DFU development. Consistent with a previous observation [48], CRP was up-regulated in the DFU patient samples. Moreover, the lower wound healing rate in DFU patients is reported to be related to higher baseline CRP levels [49]. We validated a significantly higher level of serum LRG1 in DFU patients. LRG1 is expressed during granulocyte differentiation and is involved in protein-protein interaction, signal transduction, and cell adhesion and development [50]. Consistent with our findings, Liu et al. reported elevated levels of LRG1in serum and wound tissue of DFU patients, which was partially caused by the increased infiltration of immune cells, including neutrophils and macrophages in diabetic wounds [51]. Furthermore, LRG1 was reported as an emerging therapeutic target for the treatment of vascular dysfunction, which plays a vital role in DFU development and impedes wound healing [51].

The serum complement system not only acts as a chief component of innate immunity but also plays a role in enhancing adaptive immune responses [52]. It is notable that the two cellular processes analyzed by GO were ‘response to stimulus’ and the ‘immune system process.’ Adaptive humoral immunity also plays a major role in DFU with high serum immunoglobulin lambda-like and immunoglobulin heavy chain constant regions such as IGLL5, IGLC2, IGLV3-25, IGHV3-33, and IGHA1. High serum levels of IGHA1 in DFU patient serum were verified. IGHA1 is produced by B lymphocytes and is involved in humoral immunity [53], which may serve both to defend against localized infection and prevent the access of foreign antigens to the immune system [54].

### Impaired factors of angiogenesis and vessel development in DFU

Adipose tissue significantly contributes to systemic inflammation, even under the context of potent pro-inflammatory stimuli like LPS. The major adipocyte-derived secretory protein COL6A3 is up-regulated in metabolic syndrome disorders such as diabetes [55,56]. COL6A3 acts directly through macrophage accumulation, leading to inflammation and insulin resistance, and possibly contributing to a worse metabolic profile [56].

The other identified up-regulated protein identified in DFU patient serum was DAG1. DAG1 is a laminin 1 receptor that negatively regulates angiogenic processes [57]. The up-regulation of DAG1 potentially causes the angiogenesis disorder seen in DFU.

With respect to ECM-related angiogenesis and vessel development, we also identified some down-regulated proteins, such as fibronectin (FN1), integrin β1 (ITGB1), laminin subunit gamma 1 (LAMC1), and tenascin X (TNXB). FN1 is a glycoprotein that mediates cell adhesion, growth, differentiation, and migration, all of which are involved in host defense, blood coagulation, and wound healing [58]. ITGB1 is a subunit for FN1, laminin, and collagen receptors [59]. Several studies have reported that FN1 was an important angiogenic factor [60,61]. ITGB1 is involved in endothelial development partially via controlling VE-cadherin localization and blood vessel stability [62]. The down-regulation of FN1 and ITGB1 suggests an angiogenesis deficiency and impaired wound healing in DFU. Blood vessels in TNX-deficient patients tend to be hypotonic rather than elastic [63]. TNXB plays several key roles that significantly impact the ECM and the structure of vessels. In summary, these down-regulated ECM-related proteins (FN1, ITGB1, TNXB) could be related to disturbances in vessel development during wound healing.

Based on the findings that the DEPs in DFU serum are involved in ECM-activation, immune response, and coagulation cascades, as well as angiogenesis and vessel development. The upregulated serum LRG1 [51], CD5L [38], IGHA1 [54], LBP [34], and CRP [46] were evidenced to participate in the wound-healing process. Thus, the aforementioned DEPs will potentially serve as treatment target for diabetic complications. We will further initiate individual patient cohort analysis of the correlation between the severity of DFU and the expression patterns of the identified DEPs reported herein.

## Conclusion

In this study, we identified DEPs in the serum of DFU patients. ECM-associated activation and complement and coagulation cascades are potential mechanisms involved in the pathogenesis of DFU. LRG1, CD5L, CRP, IGHA1, and LBP were quantitatively verified as DEPs. These candidates will serve as potential treatment targets for DFU. Further investigations are needed to verify and develop the application of these proteins in clinical practice.

## Supplementary Material

Supplemental Material

## Highlights

- 173 DEPs in DFU serum.
- Upregulated DEPs related to immune response and coagulation cascades.
- LRG1, CD5L, CRP, IGHA1, and LBP increase in DFU serum.
